# Prediction of Cardiac Mechanical Performance From Electrical Features During Ventricular Tachyarrhythmia Simulation Using Machine Learning Algorithms

**DOI:** 10.3389/fphys.2020.591681

**Published:** 2020-11-24

**Authors:** Da Un Jeong, Ki Moo Lim

**Affiliations:** ^1^Computational Medicine Lab, Department of IT Convergence Engineering, Kumoh National Institute of Technology, Gumi, South Korea; ^2^Computational Medicine Lab, Department of Medical IT Convergence Engineering, Kumoh National Institute of Technology, Gumi, South Korea

**Keywords:** ventricular tachyarrhythmia, computational study, mechanical performance, electrical instability, support vector regression, artificial neural network

## Abstract

In ventricular tachyarrhythmia, electrical instability features including action potential duration, dominant frequency, phase singularity, and filaments are associated with mechanical contractility. However, there are insufficient studies on estimated mechanical contractility based on electrical features during ventricular tachyarrhythmia using a stochastic model. In this study, we predicted cardiac mechanical performance from features of electrical instability during ventricular tachyarrhythmia simulation using machine learning algorithms, including support vector regression (SVR) and artificial neural network (ANN) models. We performed an electromechanical tachyarrhythmia simulation and extracted 12 electrical instability features and two mechanical properties, including stroke volume and the amplitude of myocardial tension (ampTens). We compared predictive performance according to kernel types of the SVR model and the number of hidden layers of the ANN model. In the SVR model, the prediction accuracies of stroke volume and ampTens were the highest when using the polynomial kernel and linear kernel, respectively. The predictive performance of the ANN model was better than that of the SVR model. The prediction accuracies were the highest when the ANN model consisted of three hidden layers. Accordingly, we propose the ANN model with three hidden layers as an optimal model for predicting cardiac mechanical contractility in ventricular tachyarrhythmia. The results of this study are expected to be used to indirectly estimate the hemodynamic response from the electrical cardiac map measured by the optical mapping system during cardiac surgery, as well as cardiac contractility under normal sinus rhythm conditions.

## Introduction

Recent studies on tachyarrhythmia have focused on identifying mechanisms for the development and maintenance of tachyarrhythmia by analyzing the action potential duration (APD; Lopez-Perez et al., 2019), the dominant frequency of electrical excitability (Ng et al., 2006; Hwang et al., 2016), phase singularity of reentrant waves (Mark-Anthony et al., 2001; Rantner et al., 2007; Umapathy et al., 2010; Hwang et al., 2016), and filaments of phase singularity (Clayton and Holden, 2002; Pathmanathan and Gray, 2015; Christoph et al., 2018). APD reflects the abnormalities at the tissue level as well as the cellular level. Especially, the dispersion of APD observed during the ventricular tachyarrhythmia conditions is changeable according to the pacing cycle length, pacing sites, and pacing history (Gizzi et al., 2013). The dominant frequency is the frequency band where cardiomyocytes generate the membrane potential signal with the highest power energy and can predict the degree of asynchronous excitation of the heart and repetition of the resting period (Stewart et al., 1992; Hwang et al., 2016). Phase singularity refers to topological defects in the center of reentrant waves during tachyarrhythmia (Iyer and Gray, 2001), and filaments of phase singularity represent the line of wave break inside the heart tissue where the rotating excitation waves collapse (Mironov et al., 1996; Clayton et al., 2006).

This electrical information, reflecting the degree of electrical instability due to reentrant waves, can affect asynchronous contraction during tachyarrhythmia. Among the electrical features used in the arrhythmia research, APD is used to determine the likelihood of arrhythmia through APD dispersion or APD restitution, which is a correlation with the previous diastolic interval (Gizzi et al., 2013; Sato and Clancy, 2013). Cherry and Fenton (2011) observed that tissue boundaries and geometry can cause APD dispersions in cardiac tissues with the potential of conduction block and arrhythmia development. Christoph et al. (2018) succeeded in deriving electrical and mechanical phase singularities during ventricular fibrillation through the electro-anatomical mapping system combined with high-resolution four-dimensional ultrasound. They found that electrical and mechanical phase singularities have similar properties, including topological charge, structure, dynamics, and lifespan, and suggested that the spatial-temporal electrical and mechanical systems have an inseparable relationship.

Cardiac contraction occurs asynchronously with electrical excitability due to reentry in the myocardial tissue of tachyarrhythmia (Kuklik et al., 2017). Asynchronous contraction of the ventricles by reentrant waves results in the ventricles consuming about 80% more oxygen than that consumed during normal heart contractions. However, myocardial contractile activity in tachyarrhythmia is unable to supply blood flow to meet these metabolic demands, resulting in a decrease in the volume of the ventricles to the point of end-systolic volume. Therefore, during tachyarrhythmia, blood flow through the ventricles decreases (Pansegrau and Abboud, 1970; Buckberg and Hottenrott, 1975). It is difficult to quantitatively confirm these mechanical behaviors due to mechanical irregularity caused by electrical instability. To date, no studies have sought to estimate mechanical contractility resulting from electrical instability during ventricular tachyarrhythmia.

There are many cardiac models to elucidate the mechanism of the development and maintenance of tachyarrhythmia and the resulting hemodynamic response (Ten Tusscher et al., 2009; Panfilov et al., 2010). In previous studies, we succeeded in predicting cardiac mechanical responses to various hereditary tachyarrhythmias using the electromechanical-hemodynamic coupling model developed by our research team (Lim et al., 2012, 2015; Choi et al., 2013; Heikhmakhtiar et al., 2018; Jeong and Lim, 2018; Yuniarti et al., 2018). In this study, we extracted electrical instability features from ventricular tachyarrhythmia simulation. Then, we predicted the contractile performance of the heart from the extracted electrical instability features under the assumption that there would be a correlation between the complex electrical pattern due to tachyarrhythmias and cardiac contraction. We predicted the cardiac contractile performance using support vector regression (SVR), which is commonly used for deduction of linear regression, and the artificial neural network (ANN) regression model.

## Materials and Methods

### Ventricular Tachyarrhythmia Simulation Using Cardiac Excitation-Contraction Coupling Model

**Figure 1:**
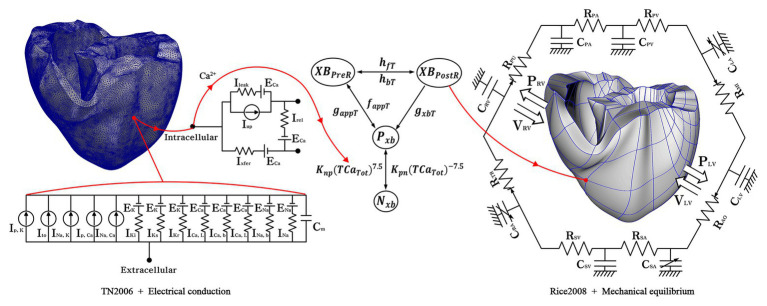
Schematic of the electromechanical model with implementation of one-way coupling in the cardiac excitation-contraction mechanism. The left side of the circuit diagram depicts a human electrophysiological ventricular model, which consists of 619,360 nodes and 3,439,590 tetrahedron elements. The electrical components of the schematic represent the current, pump, and ion exchanger from Ten Tusscher et al. (2009), which emulate the cell membrane for ion transport and the sarcoplasmic reticulum within cardiac cells. “*I*” is the ion currents, and “*E*” is the equilibrium potential of each ion; the right side depicts a human mechanical ventricular model, which is consists of 14,720 nodes and 6,210 hexahedron elements. The mechanical components represent excitation-contraction mechanism through cross-bridge of myofilaments from Rice et al. (2008) Conformations of a regulatory protein represent as non-permissive (*N_xb_*) and permissive (*P_xb_*), and the state of the myosin is denoted as *XB_PreR_* of the pro-rotated state and *XB_PostR_* of post-rotated state. *g_xbT_*, *h_fT_*, and *h_bT_* are the rate of the ATP-consuming detachment transition, the forward transition and, the backward transition, respectively. *f_aapT_* refers to the cross-bridge attachment rate of the changeover to the first strongly bound state, and *g_aapT_* refers to its reverse rate. *K_np_* and *K_pn_* denote transition rates; *K_np_*(*TCa_Tot_*)^7.5^ and *K_pn_*(*TCa_Tot_*)^−7.5^ are the transition rates of the nonpermissive to permissive (forward) and the permissive to non-permissive (backward), respectively. The mechanical model is combined with the circulatory model using the coupling method suggested by Gurev et al. (2011) “*R*” and “*C*” are the resistance and compliance of the cardiac circulatory system, respectively (For more details, see the text).

Electromechanical simulations with cardiac excitation-contraction coupling characteristics were performed to implement various electrical patterns due to tachyarrhythmias and the resulting mechanical contractions. We simulated ventricular tachyarrhythmia using a three-dimensional human ventricular model, an ion channel model suggested by Ten Tusscher (2004), and an electroconductive characteristic equation of myocardial cells, as shown on the left side of Figure 1. The mechanism of exchange of ions through the cell membrane was implemented using the lumped-parameter electrical circuit structure shown in Figure 1. In the lumped-parameter electrical circuit, “*I*” and “*R*” represent the ion channel current and the ion channel resistance, respectively. “*C*” represents the membrane capacitance (Ten Tusscher, 2004).

To simulate cardiac mechanical behavior and the hemodynamic response of ventricles in tachyarrhythmia, excitation-contraction coupling simulation was performed using calcium information extracted from the electrophysiological ventricular tachyarrhythmia simulation results. The transient calcium information was extracted using the calcium dynamic equation suggested by Ten Tusscher and Panfilov (2006), which was implemented to express the calcium cycling mechanism (calcium-induced calcium-released, CICR). Then, we used the extracted calcium as the input for the ventricular mechanical contraction simulation. The calcium information of the high-resolution electrophysiological model is transmitted to the low-resolution mechanical model as depicted in Figure 1. Here, the node information of the mechanical model is transmitted by integrating the calcium information from the adjacent nodes of the electrical model. The transmitted calcium was used to build the Troponin-C for the cross-bridge formation. For mechanical contraction simulation, the three-dimensional Hermite ventricular model, the cross-bridge equations of the myocardium suggested by Rice et al. (2008), and the circulation dynamics model were used to simulate ventricular tissue contraction and calculate the mechanical response in the ventricular tachyarrhythmia (refer to Supplementary Material for detail; Kerckhoffs et al., 2007; Rice et al., 2008; Gurev et al., 2011). In the lumped-parameter circulation circuit, C and R denote the compliance and the resistance. “P” and “V” refer to the pressure and volume, respectively; PA, the pulmonary artery; PV, the pulmonary vein; LA, the left atrium; LV, the left ventricle; MI, the mitral valve; AO, the aortic valve; RA, the right atrium; SA, the systemic artery; SV, the systemic vein; TR, the tricuspid valve; PU, the pulmonary valve.

To implement ventricular tachyarrhythmia with various electrical patterns, we used three methods in combination. First, we sequentially increased the electrical conductance of potassium channels (*g_Ks_* and *g_Kr_*) in the ventricular tissue cells model 2-, 4-, 6-, 8-, 10-, 20-, 30-, 40-, 60-, 80-, and 100-fold from the normal range. Second, in the S1–S2 protocol, which is a method for generating reentry in tachyarrhythmia simulation, we applied the S2 stimulus position to four positions; the whole left ventricle, the lower part of the left ventricle, the whole right ventricle, and the lower part of the right ventricle. From the combination of first and second methods, we performed 96 cases of ventricular tachyarrhythmia simulations. Lastly, we implemented ventricular tachyarrhythmia induced by the following five genetic mutations KCNQ1 S140G (Kharche et al., 2012; Jeong and Lim, 2018), KCNQ1 V241F (Ki et al., 2014; Heikhmakhtiar et al., 2018), KCNQ1 G229D (Hasegawa et al., 2014; Yuniarti et al., 2018), hERG L532P, and hERG N588K (Loewe et al., 2014). Thereby, we simulated 20 cases of ventricular tachyarrhythmia according to the types of mutations. All of the simulations are conducted during 10 s after re-entry was generated.

### Extraction of Electrical and Mechanical Features From Ventricular Tachyarrhythmia Simulation

To make the machine learning model for predicting the mechanical contractions during ventricular fibrillation, we obtained the electrical indices on the tetrahedral elements and the mechanical indices on the cubic Hermite elements of the three-dimensional ventricular models mentioned in Section Ventricular Tachyarrhythmia Simulation Using Cardiac Excitation-Contraction Coupling Model. Then, we averaged the electrical and mechanical indices in each node of the mesh and used these as the representative indices for training and testing the models. The electrical features were extracted by quantifying the electrical instability caused by reentrant waves in tachyarrhythmia. The extracted electrical instability features are the APD, dominant frequency of the excitation waves, phase singularity of reentry, and filaments of the phase singularities (refer to Supplementary Material for more detail). Finally, we extracted 12 electrophysiological features from the electrical instability in ventricular tachyarrhythmia (Table 1).

**Table 1:** Extracted features and outputs.

Features	Definitions	Average	*SD*	Max	Median	Min
APD	Action potential duration (ms)	121.5	43.4	237	111	69
Wavelength	Length of propagating wave (cm)	8.4	3.0	16.2	7.6	4.8
Rotation_rate	Rotational speed of reentrant wave (cm/s)	5.7	1.1	7.3	5.6	3.4
DF_mean	Mean dominant frequency on the ventricular mesh (Hz)	5.7	1.0	7.1	5.8	3.5
DF_std	Standard deviation of dominant frequency on the ventricular mesh (Hz)	0.1	4.6E-2	0.3	9.7E-02	1.1E-03
DF_peakP_mean	Mean of power spectral density at dominant frequency	0.1E-03	4.6E-05	0.2E-04	1.1E-04	4.5E-07
DF_peakP_std	Standard deviation of power spectral density at dominant frequency	2.1E-05	2.5E-05	2.7E-04	1.7E-05	3.4E-08
PS	Average number of phase singularities	48	28	119	48	5
PS_std	Standard deviation of number of phase singularities	8.0	6.1	47.3	7.2	1.0
Filament	Average number of filaments	13,782	19,321	138,142	9,648	413
Filament_std	Standard deviation of number of filaments	4,339.4	5,590.4	38,546.6	2,563	276.8
Filament/PS	Ratio of the average number of phase singularities to the average number of filaments (Length of filament)	252.5	232	1,428	192	85
Outputs	Definitions	Average	SD	Max	Median	Min
SV (mL)	Average stroke volume during meaningful periods	0.3	0.5	2.7	0.2	0
Tension-SD (kPa)	Average of amplitude of myocardial tension during the ventricular tachyarrhythmia	0.4	0.3	1.6	0.3	4.1E-02

Mechanical contractility during ventricular tachyarrhythmia was quantified by stroke volume and ampTens. Through tachyarrhythmia simulation, we defined the meaningful ejection period during which blood flowed in and out of the left ventricle by changing the volume of the left ventricle during tachyarrhythmia (Jeong and Lim, 2018). We then obtained stroke volume by averaging the difference between the end-diastolic and the end-systolic volume in the meaningful ejection period during ventricular tachyarrhythmia. We measured the end-diastolic volume, which is the volume right before ventricular volume maximally increases and then decreases, and the end-systolic volume, which is the volume right before ventricular volume minimally decreases and then increases.

Myocardial tension developed in the ventricular tissue cells was calculated using the extracted calcium information from the ventricular tachyarrhythmia simulation and the cross-bridge dynamic model of myocardial tissue as suggested by Rice et al. (2008). To quantify tension reflecting the mechanical irregularity of ventricular tissue, we averaged the standard deviations of myocardial tensions during tachyarrhythmias in all myocardial cells and used it as a feature of myocardial tension (refer to the Supplementary Material).

### Construction of the Regression Model

A conventional SVR model and the ANN regression model were used to predict ventricular mechanical contractility using extracted electrical features from ventricular tachyarrhythmia simulation. MinMaxScaler was used to prevent overfitting and underfitting due to the unit difference between each electrical feature before applying them to the regression model. The SVR model uses a linear kernel that can reflect the linear relationship between electrical and mechanical features, and a polynomial and radial basis function (RBF) kernel that can reflect nonlinear relationships. The regularization parameter of the three kernels was set to 100. In the RBF kernel, the kernel coefficient gamma was used as the inverse of the number of electrical features.

We compared the predictive performance of cardiac mechanical contractility in the ANN regression model according to the number of hidden layers. Each hidden layer consisted of six neurons, and the weights were initialized using the “uniform” method. The activation functions used were the “ReLU” function for the hidden layer and the “linear” function for the output layer to implement the regression model. To train the regression model, we monitored the mean squared error (MSE) and used the “Adam” as the optimization algorithm of error.

Of the total data, 70% was used to train the regression model and 30% was used to test the regression model created. The train set and the test set were evenly distributed after randomly splitting. The final regression model was selected using 10-fold cross-validation to avoid overfitting or underfitting of the regression model due to the small number of data. The predictive performances of the SVR model and the ANN regression model were evaluated using the determination coefficient (*R*^2^) and the MSE, respectively.

## Results

### Ventricular Tachyarrhythmia Simulation Results

Cardiac mechanical contractility in the development of ventricular tachyarrhythmia, which was implemented through electromechanical simulation, was quantified in terms of stroke volume and the amplitude of myocardial tension (ampTens). To predict contractility from the electrical properties observed during ventricular tachyarrhythmia, we extracted 12 electrophysiological features including APD, the electrical wavelength in the myocardial tissue, the rotational rate of the reentrant wave, the dominant frequency band of electrical excitation, the power spectrum density at that dominant frequency, the phase singularity, and the filament of phase singularities during tachyarrhythmia (Supplementary Figures S1–S5). The statistics of ventricular electrical and mechanical responses quantified from 116 tachyarrhythmia simulations are shown in Table 1.

### Prediction of Cardiac Mechanical Performance Using Support Vector Regression

**Figure 2:**
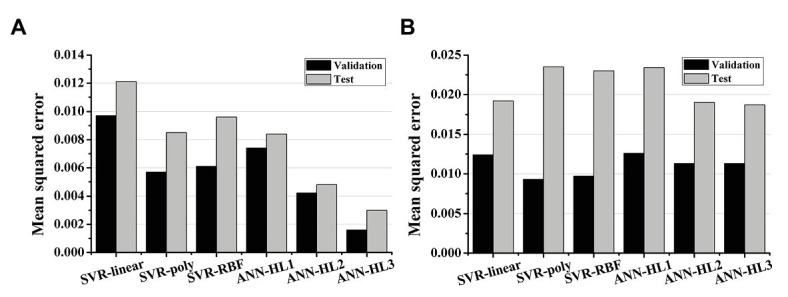
Mean squared error of the regression models. **(A)** The prediction performances of stroke volume and **(B)** myocardial tension using support vector regression (SVR) models and artificial neural network (ANN) models; SVR models have a linear kernel, polynomial kernel, and RBF kernel; the number of hidden layers (HL) in the ANN model increases from 1 to 3.

**Figure 3:**
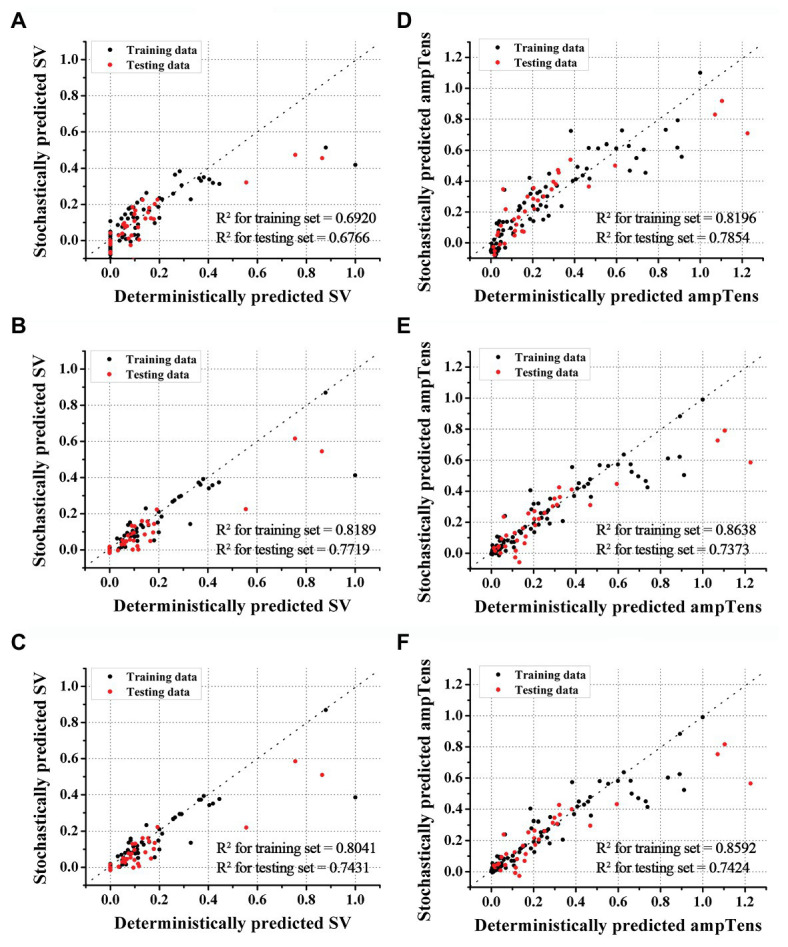
Accuracies of SVR models. Accuracies of stroke volume (SV) prediction using SVR with linear **(A)**, polynomial **(B)**, and RBF kernels **(C)**. Accuracies of myocardial tension (ampTens) prediction using SVR with linear **(D)**, polynomial **(E)**, and RBF kernels **(F)**, respectively.

Figures 2, 3 show the results of estimating stroke volume and ampTens using a commonly used SVR model. We compared the prediction performance of ventricular contractility according to the kernel type [linear kernel, RBF kernel (known also as the Gaussian kernel), or polynomial kernel]. The MSE of the testing stroke volume dataset was the lowest, and the prediction accuracy of the testing stroke volume dataset was the highest in the SVR model using the polynomial kernel (Figure 2A). In the SVR model with the polynomial kernel, the determination coefficients (*R*^2^) of stroke volume were 0.8189 for the training dataset and 0.7719 for the testing dataset (Figure 3B). The MSE of the predicted stroke volume was 0.0057 and 0.0085 in the training and testing dataset, respectively, which tended to overfit. The prediction accuracy of stroke volume was the lowest when using the SVR model with a linear kernel. The *R*^2^ of the predicted stroke volume through the SVR model using a linear kernel was 0.6920 for the training dataset and 0.6766 for the testing dataset (Figure 3A). The MSEs between the predicted stroke volume from the SVR model with a linear kernel and calculated stroke volume through the ventricular tachyarrhythmia simulation were 0.0097 and 0.0121 in the training and testing dataset, respectively, in which overfitting was reduced compared to that in the SVR model with the polynomial kernel. The tendency to overfit the training dataset was greatest in the SVR model using RBF kernel. The MSEs and *R*^2^ of predicted stroke volume through the SVR model with an RBF kernel were 0.0061 and 0.8041 in the training set, and 0.0096 and 0.7431 in the testing set, respectively (Figure 3C).

The predictive performance of ampTens predicted using SVR models was better when using the kernel considering the linear relationship (linear kernel) than the kernel considering the nonlinear relationship (RBF kernel and polynomial kernel) between the electrical and mechanical properties (Figure 2B). The predictive accuracy of the training dataset for ampTens was the highest in the SVR model with a polynomial kernel (MSE = 0.0093 for the training set, and 0.0235 for the testing set) as well as the stroke volume, but the predictive accuracy of the testing dataset for ampTens was the highest in the SVR model with a linear kernel (MSE = 0.0124 for the training set, and 0.0192 for the testing set). As with the prediction of stroke volume, the predictive performance of SVR models with three kernels tended to overfit the training dataset of ampTens. The tendency of overfitting was the highest in the SVR model using a polynomial kernel and the lowest in the SVR model using a linear kernel.

The *R*^2^ of predicted ampTens of the SVR model with a linear kernel was 0.8196 in the training set and 0.7854 in the testing set (Figure 3D). The prediction accuracy of the ampTens for the training set was the highest with an *R*^2^ of 0.8638, but for the testing set, the accuracy was the lowest with an *R*^2^ of 0.7373, which means that the tendency of overfitting was the highest (Figure 3E). The *R*^2^ of predicted ampTens through the SVR model using an RBF kernel was 0.8592 for the training set and 0.7424 for the testing set, which was almost similar to the prediction results of the SVR model with a polynomial kernel; no significant differences were found between them (Figure 3F).

### Prediction of Cardiac Mechanical Performance Using Artificial Neural Network Regression

**Figure 4:**
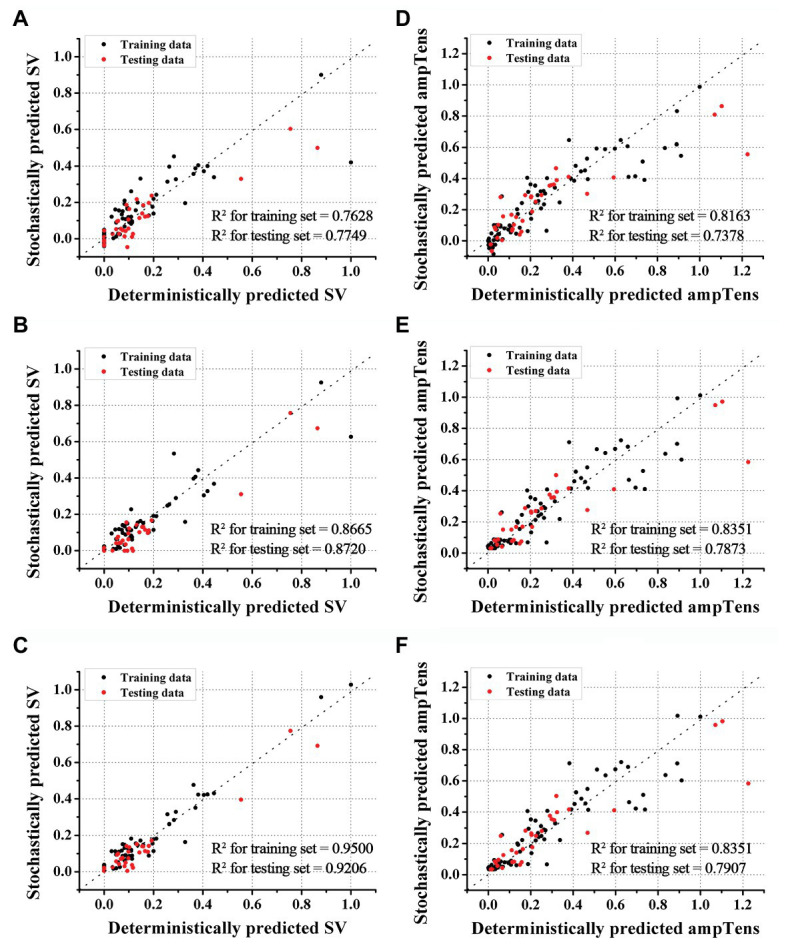
Accuracies of ANN regression models. Accuracies of stroke volume (SV) prediction using ANN models with one **(A)**, two **(B)**, and three **(C)** hidden layers. Accuracies of myocardial tension (ampTens) prediction using ANN with one **(D)**, two **(E)**, and three **(F)** hidden layers.

We predicted the stroke volume and ampTens using the ANN regression model and compared its predictive performance with that of the commonly used SVR models (Figures 2, 4). Furthermore, the predictive performance of the ANN regression model was compared according to the number of hidden layers (1, 2, or 3 hidden layers). The prediction accuracy of stroke volume using the ANN regression model was higher than that of the SVR model, regardless of the number of hidden layers. The predictive performance of stroke volume was best in the ANN regression model with three hidden layers, and the prediction accuracy decreased as the number of hidden layers decreased. As with the SVR models, there were tendencies to overfit the training set in the ANN regression models. Overfitting was greatest in the ANN regression model with three hidden layers, which also had the best predictive performance for stroke volume (MSE = 0.0016 for training data and 0.0030 for testing data), and lowest in the ANN regression model with two hidden layers (MSE = 0.0042 for training data and 0.0048 for testing data). Using the ANN regression models with two and three hidden layers, *R*^2^ of stroke volume for training data and testing data was 0.8720 and 0.9206, respectively (Figures 4B,C). The MSE of stroke volume predicted by the ANN model with one hidden layer was 0.0074 (*R*^2^ = 0.7628) for the training set and 0.0084 (*R*^2^ = 0.7749) for the testing set (Figure 4A).

As with stroke volume, the accuracy of predicted ampTens through the ANN regression model increased as the number of hidden layers increased. In all three ANN regression models, the prediction results of ampTens were overfitted to the training set. The degree of overfitting was the lowest in the ANN regression model with three hidden layers, which also had the best predictive performance (MSE = 0.0113 for the training set and 0.0187 for the testing set). The *R*^2^ of predicted ampTens from the ANN regression model consisting of three hidden layers was 0.8351 for the training set and 0.7907 for the testing set (Figure 4F). In the ANN regression model with one hidden layer, the *R*^2^ of predicted ampTens was 0.8163 (MSE = 0.0126) for training data and 0.7378 (MSE = 0.0234) for testing data, which were markedly overfitted (Figure 4D). The prediction accuracy for ampTens training data of the ANN regression model consisting of two hidden layers was similar to that of the ANN regression model with three hidden layers (*R*^2^ = 0.8351 and MSE = 0.0113), but the predictive performance for ampTens testing data was lower (*R*^2^ = 0.7873 and MSE = 0.0187, Figure 4E).

## Discussion

We predicted ventricular mechanical performance during ventricular tachyarrhythmia using the conventional SVR models and ANN regression models. The ventricular mechanical performance was predicted by the 12 electrical instability features extracted from 116 ventricular tachyarrhythmia cases implemented through excitation-contraction coupling simulations. The main findings of the study were:

1. In the SVR models, the predictive accuracy of stroke volume was the highest when using the polynomial kernel (*R*^2^ = 0.7719, and MSE = 0.0085), and the predictive accuracy of ampTens was the highest when using the linear kernel (*R*^2^ = 0.7854 and MSE = 0.0192).
2. In predicting the cardiac mechanical contractility (both stroke volume and ampTens), the prediction performance of ANN regression models was better than that of SVR models.
3. In the ANN model, the prediction accuracy of mechanical contractility during ventricular tachyarrhythmia increases as the depth of the hidden layer increases; thus, the best performance was found in the model with three hidden layers.

The *K*^+^ current increases and decreases with changes in the electrical conductance of *K*^+^ channels, which consists of a transient outward *K*^+^ channel, a slow-delayed rectifier *K*^+^ channel, and a rapid-delayed rectifier *K*^+^ channel. Changes in the electrical conductance of *K*^+^ channels induce changes in *K*^+^ current and produce various electrical patterns in ventricular tachyarrhythmia. An increased *K*^+^ current decreases the APD of cardiomyocytes, thereby facilitating the reentry of the electrical excitation wave and easily causing tachyarrhythmia (Ravens and Cerbai, 2008). In this study, the electrophysiology model we used was based on Ten Tusscher’s (2004) human ventricular model. In this model, they considered the heterogeneity of ventricular tissue and suggested the different values for potassium channel conductances such as *g_Ks_*, *g_kr_*, and *g_to_*. The transient outward *K*^+^ currents strongly affect phase 1 (rapid repolarization) during action potential generation (Shih, 1994) but have less impact on the APD. Thereby, we changed the electrical conductances (*g_Ks_* and *g_Kr_*) of the slow-delayed rectifier *K*^+^ channel and the rapid-delayed rectifier *K*^+^ channel to implement ventricular tachyarrhythmia conditions with various electrical patterns.

However, many researchers suggested that, in the initiation of ventricular tachyarrhythmia and the development of arrhythmias, action potential amplitude is more important than the APD and presented the need to see not only the APD but also action potential amplitude in the study of tachyarrhythmia. Some of the fibrillation is occurred by the action potential amplitude alternans, not APD alternans, thereby, APD does not reflect the action potential amplitude alternans (Gizzi et al., 2013; Chen et al., 2017). In this study, we obtained the dominant frequency by Fourier transforming the change in action potential overtime when tachyarrhythmia occurs, and it is the maximum power on the spectral density and contains the information of action potential amplitude. The stroke volume during ventricular tachyarrhythmia was obtained by measuring the changes of the left ventricular volume according to the in and out of the blood movement. However, the myocardial tension was obtained by calculating the standard deviation of the myocardial tension from the whole nodes of the human ventricular models according to the Rice model (Supplementary Figures S4, S5). That is, while the stroke volume is a global metric, the ampTens comes from the mathematic model. However, stroke volume is affected by the ampTens, and they have a proportional correlation as follows: (Myocardial tension/afterload) ∝ stroke volume. This can also be confirmed by the Pearson correlation coefficient in Supplementary Figure S6.

We simulated the 116 episodes of ventricular tachyarrhythmias and calculated 12 electrical features from the electrophysiological simulations. While APD was 236 ms in the control condition without any changes of conductance, APD decreased to 69 ms in 100-fold *g_Ks_* and 2-fold *g_Kr_* condition, which was the state that ventricular fibrillation is the most severe. Clinically, it was reported that the APDs of patients with the V241F mutation or the KCNJ2 E299V mutation were about 72–76 ms, which is similar to APD in 100-fold *g_Ks_* condition (Deo et al., 2013; Heikhmakhtiar et al., 2018). This result is corresponding to Kappadan et al. (2020) findings that the shorter APD is, the faster and more complex ventricular fibrillation became.

The various tachyarrhythmia conditions we implemented are including both tachycardia and fibrillation conditions. Dominant frequency was obtained by performing frequency analysis using the FFT during whole ventricular tachyarrhythmia conditions and determining the frequency band at the highest power spectral density. The point of dominant frequency is at the rotor of reentry. Therefore, if reentrant break-up occurs, the number of reentrant rotors increases and dominant frequency appears heterogeneous Supplementary Figure S2a However, if reentry breakup does not occur and is stably sustained, the dominant frequency in the heart tissue may have homogenous patterns as shown in Supplementary Figure S2b.

It is possible to mathematically express and simulate physiological changes that occur as a result of drugs, genetic mutations, and various heart diseases through the electromechanical finite element model, which can mimic the functional characteristics of ion channels, buffers, and transporters (Chang and Trayanova, 2016; Niederer et al., 2019). In the last few decades, the electromechanical finite element model has evolved rapidly with regards to the modeling of heart shapes as well as in the accuracy and precision of results to successfully predict cardiac dynamics (Trayanova, 2011; Pierre et al., 2012). Deo et al. (2013) simulated electrophysiological changes when KCNJ2 E229V genetic mutations were expressed using a rabbit three-dimensional ventricular model, based on Ohara Rudy’s ventricular cell model, and verified the results using experimental data (Deo et al., 2013). Pathmanathan and Gray (2015) simulated ventricular fibrillation using a high-resolution rabbit ventricle model and observed movement of the filaments in reentrant waves. Ten Tusshcer and Nash implemented ventricular fibrillation through clinical trials and human heart modeling and observed changes in APD, phase singularities, and filaments during ventricular fibrillation (Ten Tusscher et al., 2009). Furthermore, we have successfully estimated mechanical and hemodynamic responses under tachyarrhythmias caused by hereditary mutations (Heikhmakhtiar et al., 2018; Jeong and Lim, 2018; Yuniarti et al., 2018), as well as under conditions resulting from heart auxiliary devices such as the ventricular assist device, and cardiac resynchronization therapy (Lim et al., 2012; Choi et al., 2013; Heikhmakhtiar and Lim, 2018; Park et al., 2018). The electrical and mechanical features were generated by the excitation-contraction coupling simulation validated by renowned research groups and journals.

In the real world, it is hard to measure cardiac contractility during ventricular tachyarrhythmias. Furthermore, in a cardiac arrhythmia surgery such as ablation, the electrical state of the heart is checked mainly through the electro-anatomical mapping system, but, the hemodynamic response cannot be seen. In this paper, we simulated the ventricular tachyarrhythmia situations using the deterministic models and then, predicted the hemodynamic response as the mechanical performance under the various pathologic status and mutation conditions from the electrical features using the stochastic models, which is the machine learning model. Therefore, we expect that the results of this study can be used to indirectly estimate the hemodynamic response from the electrical heart map measured by the electro-anatomical mapping system during cardiac surgery, not only the heart contractility under the normal sinus rhythm condition. Therefore, we expect that the results of this study can be used to indirectly estimate the hemodynamic response from the electrical heart map measured by the optical mapping system during cardiac surgery.

Advanced studies have focused on the electrophysiological effects of myocardial tissue contraction caused by reentrant waves using a mechanoelectrical model (Hu et al., 2013). Nash and Panfilov (2004) and Hu et al. (2013) showed that deformed shrinkage patterns can increase the duration of reentrant waves and cause the collapse of reentrant waves due to activation of *K*^+^ conductive stretch-active cells and reduction of the APD (Van Wagoner, 1993). They were able to show this through a two-dimensional electromechanical coupling model, which combines mechanical and electrical properties of cardiac tissue (Nash and Panfilov, 2004; Hu et al., 2013). Panfilov et al. (2010) found during the ventricular tachyarrhythmia that the stationary reentrant wave can break up into several smaller waves through the mechanoelectrical feedback of stretch-activated channels. In this study, however, we aimed to predict the mechanical response from electrical patterns generated during ventricular tachyarrhythmia. Therefore, we did not take into account the occurrence of reentrant waves due to tissue contraction and the resulting electrophysiological changes in consideration of the purpose of the study and the efficiency of computing resources. Furthermore, mechanical contractility and mechanical instability were decreased in proportion to the electrical instability (Supplementary Figures S1–S6). Hence, even if mechanoelectrical feedback is considered, the electrical instability is expected to become more unstable in proportion to the mechanical discoordination, which is not expected to affect the overall conclusion of this study. In future work, we are planning to predict the cardiac contractility considering the mechanoelectrical feedback. Besides, the electromechanical model used in this study was implemented without taking into account other factors such as anatomy, hypertrophy, expansion, or electromechanical delay. Under the ventricular tachyarrhythmia conditions, these factors can play an important role but, our simulation data does not reflect these factors. To get the robustness, our ANN model needs to be validated using the electrical activity features that affect hemodynamics.

The SVR calculates the distance of the samples used in the prediction of the regression model using the vector dot product to estimate the optimal model. If the samples are linearly distributed, good prediction performance can be achieved even with a linear kernel. However, in most cases where samples are nonlinearly distributed, using a linear kernel can reduce performance by increasing prediction error. Therefore, the kernel should be selected properly according to the distribution of the samples. To determine the relationship between these samples, we performed Pearson’s correlation analysis and confirmed the relationship between the 12 electrical instability features and mechanical properties extracted from the ventricular tachyarrhythmia simulation (Supplementary Figure S6). Some of the 12 extracted electrical features had strong positive or negative linear relationships with mechanical contractility, while others had a weak linear relationship with mechanical contractility. The electrical features linearly related to mechanical characteristics, APD, conduction wavelength, the rotational rate of the reentrant wave, and dominant frequency of electrical excitation (Pearson correlation coefficient >0.7) were directly related to the electrical activity of the cardiomyocytes. However, the electrical features related to electrical instability of myocardial tissue caused by reentrant waves, such as the phase singularity, filament of phase singularities, and the spatial distribution of the dominant frequency had no linear relationship with mechanical contractility.

Accordingly, we filtered the electrical features that had a linear relationship with mechanical contractility, i.e., those with a Pearson correlation coefficient of >0.5. The filtered electrical features were APD, conduction wavelength, the rotational rate of the electrical excitation, mean of dominant frequency, mean of the power spectral densities in the dominant frequency band, the variance of the number of phase singularities, and variance of the number of filaments. When using these seven electrical features, the predictive performance of the SVR model with a linear kernel was improved for the prediction of both stroke volume and ampTens (Supplementary Figure S7). The prediction accuracy of the SVR model with the linear kernel was 0.7708 (MSE = 0.0085) for the stroke volume and 0.8009 (MSE = 0.0178) for the ampTens, but it was still lower than the prediction performance using the ANN regression model. Therefore, we thought that the ANN regression model will be possible to predict mechanical performance by considering the nonlinear and complex relationship between electrical and mechanical features. However, the trained parameters from the ANN model may not have physiological meanings.

The number of hidden layers in the ANN regression model is an important factor in multilayer perceptron learning and is introduced to address problems that are difficult to solve linearly (Adrian, 1997). In a multilayer perceptron, hidden layers filter or refine the raw data and send it to the next layer to determine the decision boundary of each layer. If the number of hidden layers is too small, the learning does not work well; however, if the number of hidden layers is too high, it not only takes a long time to train the model but also may result in overfitting of the training data. Therefore, it is necessary to select the number of hidden layers according to each data set (Adrian, 1997). In this study, we compared the performance by increasing the number of hidden layers to find the optimal ANN regression model for predicting the mechanical contractility from electrical instability features extracted from ventricular tachyarrhythmia simulation. Therefore, the ANN regression model with three hidden layers showed the best performance in the prediction of both stroke volume and ampTens. We compared the predictive performance between the ANN model consisting of four hidden layers and the ANN model consisting of three hidden layers, but the performance was not enhanced and we found an increased tendency for overfitting (Supplementary Figure S8). Therefore, we propose the ANN regression model with three hidden layers as an optimal model for predicting cardiac mechanical contractility in ventricular tachyarrhythmia.

## Conclusion

In this study, we compared the performance of SVR and ANN regression models to predict the mechanical contractility from 12 electrical instability features in ventricular tachyarrhythmia. Even though every model was overfitted to the training dataset a bit, the proposed ANN model with three hidden layers was able to predict the mechanical performance with high accuracy. Every data we used in this study were obtained from the electromechanical simulation. Therefore, our proposed ANN model requires further validation using some clinical data to confirm the robustness of the model.

## Data Availability Statement

All datasets generated for this study are included in the article/Supplementary Material.

## Author Contributions

This paper is the intellectual product of the entire team. Both the authors contributed (to varying degrees) toward the analyses performed, developing the research concept, simulation design, and the simulation source code, performing the simulation, and writing of the manuscript. All authors contributed to the article and approved the submitted version.

### Conflict of Interest

The authors declare that the research was conducted in the absence of any commercial or financial relationships that could be construed as a potential conflict of interest.
