# Supplementary material for: Prediction of Cardiac Mechanical Performance From Electrical Features During Ventricular Tachyarrhythmia Simulation Using Machine Learning Algorithms
Source: Front Physiol. 2020 Nov 24;11:591681. doi: 10.3389/fphys.2020.591681 (PMC7732497; doi:10.3389/fphys.2020.591681)
Supplement: Supplementary file 1 [file Data_Sheet_1.pdf]

Supplementary Material

Prediction of Cardiac Mechanical Performance from Electrical Instability Features during Ventricular Tachyarrhythmia Simulation using Machine Learning Algorithms.

Da Un Jeong[1], Ki Moo Lim[2*]

*** Correspondence:** Ki Moo Lim: kmlim@kumoh.ac.kr

1 Electromechanical human ventricular model

The human electromechanical ventricular model with excitation-contraction coupling characteristics was used to simulate ventricular tachyarrhythmia with various electrical patterns and the resulting cardiac mechanical contraction. The 3-dimensional electrophysiological ventricular model consisted of 619,360 nodes and 3,439,590 tetrahedral elements and was implemented based on the MRI images. To consider the structural characteristics and thickness of the ventricles, we used the heterogenous human ventricular model, which was implemented according to the conductance variation; Gks = 0.392∗1.3 mS/μF and Gto = 0.073 mS/μF for the endocardium; Gks = 0.098∗2.0 mS/μF and Gto = 0.294 mS/μF for the mid-myocardium; Gks = 0.392∗1.3 mS/μF and Gto = 0.294 mS/μF for the epicardium (Ten Tusscher, 2004; Ten Tusscher and Panfilov, 2006). Each part of the ventricles was distinguished according to the location and thickness; we determined the adjacent tissues from the epicardial surface to the inside for 1mm as the epicardium, the adjacent tissues from the endocardial surface to the inside for 1mm as the endocardium and the other tissues as the mid-myocardium.

The electrical conductivity of the ventricular cell was expressed using the validated ventricular cell model and the 3-dimensional finite element analysis. The ventricular electrophysiological model was consisted of the lumped-parameter model to simulate the ion exchange mechanism through the cell membrane (Ten Tusscher, 2004; Ten Tusscher and Panfilov, 2006).

$$\frac{dV_m}{dt} = -\frac{I_{ion}+I_{stim}}{C_m} + \frac{1}{\rho_x S_x C_m}\frac{\partial^2 V}{\partial^2 x^2} + \frac{1}{\rho_y S_y C_m}\frac{\partial^2 V}{\partial^2 y^2} + \frac{1}{\rho_z S_z C_m}\frac{\partial^2 V}{\partial^2 z^2} \quad \textbf{Equation 1}$$

$$I_{ion} = I_{Na} + I_{Ki} + I_{to} + I_{Kr} + I_{Ks} + I_{Ca,L} + I_{Na,Ca} + I_{Na,K} + I_{p,Ca} + I_{p,K} + I_{Ca,b} + I_{Na,b} \quad \textbf{Equation 2}$$

where V_m is the membrane potential of the myocardial cell, t is the time, C_m is the cell membrane capacitance. “I” denotes the current: I_{ion} is the sum of transmembrane ionic currents; I_{stim} is the current due to the external stimulus; I_{Na} is the Na^+ current; I_{Ki} is the inward rectifiers K^+ current; I_{to} is the transient outward K^+ current; I_{Kr} and I_{Ks} are the rapid-delayed rectifier K^+ current and the slow-delayed rectifier K^+ current, respectively; $I_{Ca, L}$ is the L-type inward Ca^{2+} current; $I_{Na, Ca}$ is the Na^+-Ca^{2+} exchange current; $I_{Na, K}$ is the Na^+-K^+ exchange current; $I_{p, Ca}$ and $I_{p, K}$ are the pump currents of Ca^{2+} and K^+, respectively; and $I_{Ca, b}$ and $I_{Na, b}$ are the background currents of Ca^{2+} and K^+, respectively. “E” denotes the equilibrium potential; E_K, E_{Ca}, and E_{Na} are the equilibrium potentials of K^+, Ca^{2+}, and Na^+, respectively.

To simulate the excitation-contraction mechanism of myocardial tissues with calcium, we extracted transient calcium information from the 3-dimensional electrophysiological simulation and used it as the input for the mechanical contraction simulation (Ten Tusscher and Panfilov, 2006; Rice et al., 2008).

$$\frac{dCa_{itotal}}{dt} = -\frac{I_{Ca,L}+I_{b,Ca}+I_{p,Ca}-2I_{Na,Ca}}{2V_C F} + I_{leak} - I_{up} + I_{rel}$$ **Equation 3**

$$\frac{dCa_{srtotal}}{dt} = \frac{V_c}{V_{SR}}(-I_{leak} + I_{up} - I_{rel})$$ **Equation 4**

where Ca_{itotal} and $Ca_{srtotal}$ denote the sum of calcium in the cytoplasm and the sarcoplasmic reticulum, respectively. I_{rel} is the CICR current due to the released calcium from the junctional sarcoplasmic reticulum, I_{leak} is the leakage current from the sarcoplasmic reticulum to the cytoplasm, I_{up} is the pump current taking up calcium to the network sarcoplasmic reticulum, and I_{xfer} is the current caused by diffuse calcium between the dyadic subspace and bulk cytoplasm.

The 3-dimensional human ventricular model for mechanical contraction consists of 14,720 nodes and 230 hexahedral elements. The contraction of ventricular tissue is implemented by solving the continuum dynamic equation, assuming that the myocardium, which is a hyper-elastic, nearly incompressible material, has passive mechanical properties (Guccione et al., 1995; Usyk et al., 2002; Gurev et al., 2011). The passive mechanical property is defined by the exponential strain function (W).

$$W = \frac{C_m}{2}(e^Q - 1)$$ **Equation 5**

$$Q = b_f E_{ff}^2 + b_t(E_{rr}^2 + E_{cc}^2 + 2E_{rc}^2) + 2b_{fs}(E_{fr}^2 + E_{fc}^2)$$ **Equation 6**

$$E_{\alpha\beta} = \frac{1}{2}(\frac{\partial x^k}{\partial v^\alpha}\frac{\partial x^k}{\partial v^\beta} - \delta_{\alpha\beta})$$ **Equation 7**

where the material constant C_m is set to 2kPa, and Q is determined by the type of material that is transversely isotropic for the muscle fiber axis (Guccione and McCulloch, 1991). b_f, b_t, and b_{fs} are determined by orthotropic electrical conductivity and passive mechanical properties of the myocardium according to the laminar sheet-nominal direction and fiber orientation information and are set to 8, 2, and 4, respectively. E_{ij} is the local fiber coordinate system, Langian Green's strain. E_{ff} is fiber strain, E_{rr} is the cross-fiber in-plain strain, and E_{cc} is the radial strain. E_{rc} is a shear strain in a transverse plane, E_{fr} is a shear strain in the fiber-cross fiber plane, and E_{fc} is a shear strain in a fiber-radial coordinate plane.

The fiber and laminar sheet structural information of the ventricles is obtained from the diffusion tensor magnetic resonance image (DTMR) set. Tensor and tensor gradients were defined at each node of the finite element mesh and interpolated using Hermite interpolation to incorporate fiber and laminar sheet structures. The tensor eigenvectors from the interpolated tensor field denote the fiber and laminar sheet structured heart, as validated by Helm et al (Helm et al., 2005). The fiber orientation was allocated to each element in the electrophysiological mesh by first finding the center of the element and then applying Newton's method to map that center to the corresponding location in the mechanical mesh. In the mechanical mesh, the fiber orientation was allocated to the corresponding finite element of the electrophysiological mesh(Guccione et al., 1995; Gurev et al., 2011). To

quantitatively calculate the mechanical behaviour of ventricles in tachyarrhythmia, the hemodynamic response to the mechanical contraction of ventricles was simulated by combining the electromechanical finite element ventricular model and the circulatory dynamic model (Kerckhoffs et al., 2007; Gurev et al., 2011). The circulatory dynamic model is based on the human cardiovascular model proposed by Kerchhoffs (Kerckhoffs et al., 2007) and consists of a lumped hemodynamic model as shown on the right-hand side of Figure 4. Finally, we could calculate the ventricular blood pressure and volume, which is one of the ventricular hemodynamic responses in tachyarrhythmias.

$$\text{Pressure} = C^{-1}(t)(V - V_{rest}(t)) \quad \textbf{Equation 8}$$

$$\Delta\vec{V} = C \cdot \vec{P} = \begin{bmatrix} \Delta V_L \\ \Delta V_R \end{bmatrix} = \begin{bmatrix} C_{LL} & C_{LR}(p_L) \\ C_{RL}(p_R) & C_{RR} \end{bmatrix} \begin{bmatrix} p_L \\ p_R \end{bmatrix} \quad \textbf{Equation 9}$$

$$\boldsymbol{C_{ij} = y_v(C_{max} - C_{min}) + C_{min}} \quad \textbf{Equation 10}$$

$$\boldsymbol{V_{rest} = (1 - y_v) * \begin{bmatrix} V_{L,rest,d} & -V_{L,rest,s} \\ V_{R,rest,d} & -V_{R,rest,s} \end{bmatrix} + \begin{bmatrix} V_{L,rest,s} \\ V_{R,rest,d} \end{bmatrix}} \quad \textbf{Equation 11}$$

"C" is the time-dependent compliance matrix and consists of compliance of the left ventricle (C_{LL}) and the right ventricle (C_{RR}) calculated through the compliance when the ventricles are in the active state (C_{max}) and the passive state (C_{min}). y_v is the activation function of ventricles. "V" refers to the volume. V_{rest} is the volume when the ventricular pressure is zero. $V_{L,rest,d}$ and $V_{L,rest,s}$ are the diastolic volume and the systolic volume of the left ventricle and $V_{R,rest,d}$ and $V_{L,rest,s}$ are the diastolic volume and the systolic volume of the right ventricle, respectively.

2 Expression of mutation conditions

In this study, we conducted the simulations under the five mutation conditions (KCNQ1 S140G, KCNQ1 V241F, KCNQ1 G229D, KCNH2 (hERG) L532P, KCNH2 (hERG) N588K) to predict the mechanical response that may occur in the real-world through the electrical pattern changes due to the specific mutation, not only the artificially generated electrical patterns. We expressed the mutation conditions using the following formulas.

2.1 KCNQ1 S140G mutation condition

The KCNQ1 S140G mutation is expressed by transforming the 140th codon S of the S2 helix of KCNQ1 into codon G and is well known to induce fibrillation by increasing the electrical conductivity of the I_{Ks} channel. To mimic the changes of I_{Ks} channel due to KCNQ1 S140G mutation ($I_{Ks\text{-}S140G}$), we used the following equation (Kharche et al., 2012).

$$I_{Ks-S140G} = I_{Ks} + \varphi g_{Ks}(V_m - E'_{rev}) \quad \textbf{Equation 12}$$

where φ is a scaling factor of KCNQ1 S140G mutation and was set as 0.1 to see the effects under the intermediate mutant level based on Kharche et al.'s experiment and simulation study. E'_{rev} represents the mutable reversal potential due to the instantaneous component of the K^+ channel and was set to 75.3mV.

2.2 KCNQ1 V241F mutation condition

The KCNQ1 V241F mutation is expressed by replacing the 241th codon V with codon F in the S4 helix of KCNQ1 gene and can induce fibrillation by increasing the I_{Ks} current like the KCNQ1 S140G mutation. The I_{Ks} current under the KCNQ1 V241F mutation condition ($I_{Ks\text{-}V241F}$) was expressed as follows (Imaniastuti et al., 2014; Ki et al., 2014).

$$I_{Ks-V241F} = P_{Ks} \times \left(0.67 \times n_{fast}{}^{2} + 0.33 \times n_{slow}{}^{2}\right) \times \left(K_i \cdot e^{\delta \cdot \frac{FV_m}{RT}} - K_e \cdot e^{-(1-\delta)\cdot \frac{FV_m}{RT}}\right) \quad \textbf{Equation 13}$$

$$\bar{n}_{fast} = 0.25 + \frac{0.75}{1+e^{-(V_m+34.83766)/19.48842}} \quad \textbf{Equation 14}$$

$$\bar{n}_{slow} = \bar{n}_{fast} \quad \textbf{Equation 15}$$

$$\tau_{n_{fast}} = \left(1.8075 \times 10^{-13} \times e^{-\frac{V_m}{4.62}} + 0.02238 \times e^{\frac{V_m}{9.82}}\right)^{-1} + \left(2.02983 \times 10^{-4} \times e^{-V_m/76.68} + 8.4305 \times 10^{-4} \times e^{V_m/51.66}\right)^{-1} \quad \textbf{Equation 16}$$

$$\tau_{n_{slow}} = \left(7.58278 \times 10^{-11} \times e^{-V_m/6.81} + 0.07943 \times e^{V_m/7.86}\right)^{-1} + \left(2.02983 \times 10^{-4} \times e^{-V_m/76.68} + 8.4305 \times 10^{-4} \times e^{V_m/51.66}\right)^{-1} \quad \textbf{Equation 17}$$

$$\frac{dn_{fast}}{dt} = \frac{\bar{n}_{fast} - n_{fast}}{\tau_{n_{fast}}} \quad \textbf{Equation 18}$$

$$\frac{dn_{slow}}{dt} = \frac{\bar{n}_{slow} - n_{slow}}{\tau_{n_{slow}}} \quad \textbf{Equation 19}$$

where P_{Ks} is a conversion factor for the V241F mutation and was set to 0.25 to simulate the intermediate V241F mutation condition. n_{fast} is the fast-activation gate of the K+ channel, and $\bar{n}_{fast}$ denotes n_{fast} under the steady-state. n_{slow}_slow is the slow-activation gate of the K+ channel, and $\bar{n}_{slow}$ denotes n_{slow} under the steady-state. τ_{fast} and τ_{slow} are the time constants of the fast-activation gate and the slow-activation gate, respectively. R is a gas constant, and T is the absolute temperature. K_i and K_e are the intracellular and extracellular concentrations of K^+. δ was set to 0.15 as the asymmetric factor of the V241F mutation.

2.3 KCNQ1 G229D mutation condition

Like the KCNQ1 V241F mutation, the KCNQ1 G229D mutation is expressed in the S4 helix and occurs when the 299th codon is transformed into a D codon. The change in I_{Ks} current due to KCNQ1 G229D mutation ($I_{Ks\text{-}G229D}$) was expressed as follows (Hasegawa et al., 2014; Yuniarti et al., 2018).

$$I_{Ks-G229D} = g_{KS} \cdot \left(1 + \frac{0.6}{1+\left(\frac{3.8\times10^{-5}}{[Ca^{2+}]_i}\right)^{1.4}}\right) \cdot x_{s1} \cdot x_{s2}(V_m - E_K) \quad \textbf{Equation 20}$$

$$\frac{dx_{s1}}{dt} = \frac{(x_{s1,\infty} - x_{s1})}{\tau_{x,s1}} \quad \textbf{Equation 21}$$

$$\frac{dx_{s2}}{dt} = \frac{(x_{s2,\infty} - x_{s2})}{\tau_{x,s2}}$$ **Equation 22**

$$x_{s2,\infty} = \frac{0.85}{1 + e^{(V_m + 82.8)/41.72}}$$ **Equation 23**

$$\tau_{x,s1} = \frac{326.9 + 0.4}{2.326 \times 10^{-4} \times e^{(V_m + 119.5)/17.8} + 1.292 \times 10^{-3} \times e^{-(V_m + 281.2)/230}}$$ **Equation 24**

$$\tau_{x,s2} = \frac{5}{0.01 \times e^{(V_m - 50)/100} + 0.0193 \times e^{-(V_m + 66.54)/155}}$$ **Equation 25**

Here, x_{s1} and x_{s2} are parameters of the activated and deactivated gates, respectively. Besides, $x_{s1,\infty}$ and $x_{s2,\infty}$ are parameters when each gate is in the steady-state. $\tau_{x,s1}$ and $\tau_{x,s2}$ mean the time constants of each gate.

2.4 KCNH2 (hERG) L532P mutation condition

The KCNH2 L532P mutation and the KCNH2 N588K mutation are expressed in the human-either-go-go-related gene (hERG) and increase the I_{Kr} current to cause fibrillation. In the L532P mutation, the 532th codon L of hERG is transformed into codon P. The change of I_{Kr} current due to the KCNH2 L532P mutation ($I_{Kr\text{-}L532P}$) was expressed as follows (Loewe et al., 2014; Heikhmakhtiar et al., 2020);

$$I_{Kr-L532P} = g_{Kr-L532P} \cdot x_{r1-L532P} \cdot x_{r2-L532P} \cdot (V_m - E_K)$$ **Equation 26**

$$x_{r2-L532P} = \left[1 + exp\left(\frac{V_m - 15.54}{24.37}\right)\right]^{-1}$$ **Equation 27**

$$\frac{dx_{r1-L532P}}{dt} = \frac{x_{r1-L532P,\infty} - x_{r1-L532P}}{\tau_{x_{r1-L532P}}}$$ **Equation 28**

$$x_{r1-L532P,\infty} = \left[1 + exp\left(\frac{V_m - 9.88}{22.31}\right)\right]^{-1}$$ **Equation 29**

$$\tau_{x_{r1-L532P}} = \left[\left(\alpha_{x_{r1-L532P}} + \beta_{x_{r1-L532P}}\right)\right]^{-1}$$ **Equation 30**

$$\alpha_{x_{r1-L532P}} = 0.00025 \times \frac{V_m - 196.86}{1 - exp\left(\frac{V_m - 196.86}{-131.36}\right)}$$ **Equation 31**

$$\beta_{x_{r1-L532P}} = 7.3898 \times 10^{-5} \times \frac{V_m - 40.00}{exp\left(\frac{V_m - 40.00}{3.79 \times 10^{-6}}\right) - 1}$$ **Equation 32**

where $g_{kr-L532P}$ denotes the I_{Kr} channel conductivity due to the KCNH2 L532P mutation and was set to 0.91720 nS/pF. x_{r1} and x_{r2} are the parameters of the activated and deactivated gate. τ_{xr1} and τ_{xr2} mean the time constants of each gate.

2.5 KCNH2 (hERG) N588K mutation condition

The KCNH2 N588K mutation is expressed by the 588th codon N of hERG is transformed into codon K. The change of I_{Kr} current due to the KCNH2 N588K mutation ($I_{Kr\text{-}N588K}$) was expressed as follows (Loewe et al., 2014; Heikhmakhtiar et al., 2020);

$$I_{Kr-N588K} = g_{Kr-N588K} \cdot x_{r1-N588K} \cdot x_{r2-N588K} \cdot (V_m - E_K) \quad \textbf{Equation 33}$$

$$x_{r2-N588K} = \left[1 + exp\left(\frac{V_m - 38.65}{19.46}\right)\right]^{-1} \quad \textbf{Equation 34}$$

$$\frac{dx_{r1-N588K}}{dt} = \frac{x_{r1-N588K,\infty} - x_{r1-N588K}}{\tau_{x_{r1-N588K}}} \quad \textbf{Equation 35}$$

$$x_{r1-N588K,\infty} = \left[1 + exp\left(\frac{V_m + 16.49}{6.76}\right)\right]^{-1} \quad \textbf{Equation 36}$$

$$\tau_{x_{r1-N588K}} = \left[\left(\alpha_{x_{r1-N588K}} + \beta_{x_{r1-N588K}}\right) \times 2\right]^{-1} \quad \textbf{Equation 37}$$

$$\alpha_{x_{r1-N588K}} = 0.00030 \times \frac{V_m + 14.1}{1 - exp\left(\frac{V_m + 14.1}{-5}\right)} \quad \textbf{Equation 38}$$

$$\beta_{x_{r1-N588K}} = 7.3898 \times 10^{-5} \times \frac{V_m - 3.3328}{exp\left(\frac{V_m - 3.3328}{5.1237}\right) - 1} \quad \textbf{Equation 39}$$

where $g_{kr-N588K}$ denotes the I_{Kr} channel conductivity due to the KCNH2 N588K mutation and was set to 0.029412 nS/pF.

3 Extraction of electrical and mechanical features

To generate reentrant waves, we performed a reentry generation simulation under low conduction velocity conditions (20 cm/s) by using the S1-S2 protocol. Then, when reentrant waves were generated, all dependent variables of the cellular state at the moment the reentrant waves were stably maintained were saved and used as the initial values in the next sustained reentry simulation, which was performed at normal conduction velocity conditions (68.5 cm/s). The conduction velocity was calculated by dividing the height of the ventricular model (8.022 cm) by the time it took for the propagating waves to move from the apex to the top of the ventricles. We performed the sustained reentry simulation for up to 20 s to obtain the electrical and mechanical properties at the moment the reentrant waves reached a steady state.

We extracted 12 electrical instability features from the four electrical properties, which refer to the electrical activity of the ventricles in tachyarrhythmia: the APD, the dominant frequency, the phase singularity, and the filament. We quantified the cardiac mechanical contractility in ventricular tachyarrhythmia as the stroke volume and the amplitude of myocardial tension.

- Action potential duration (APD): APD was obtained by measuring the time taken for a myocardial cell to be repolarised to 90% after depolarisation during the pacing cycle. We averaged APDs measured during 10 s of ventricular tachyarrhythmia in the 4 regions of ventricular tissue, which are the apex, lower ventricle, central, and upper ventricle).
- Wavelength: Reentrant waves were generated under a conduction velocity of 68.5 cm/s. Therefore, the wavelength was calculated by multiplying APD and the conduction velocity.
- Rotation rate: Obtained by dividing the number of cycles during the reentry by the time when the reentrant waves were sustained.

- Dominant frequency: Obtained by performing frequency analysis of the membrane potential at each node of the ventricles and determining the frequency band at the point power spectral density was at its highest (Ng et al., 2006). Frequency analysis was performed using the Fast Fourier Transform (FFT) function of the MATLAB program, and the sampling frequency was 0.01 Hz. To use as an electrical feature for predicting the mechanical contractility, we calculated the mean and the standard deviation of the dominant frequencies obtained at all nodes.
- Peak power of the dominant frequency: Obtained from the power spectral density at the dominant frequency. We calculated the mean and the standard deviation of peak power obtained at all nodes.
- Phase singularity: To detect phase singularity of reentrant waves, the membrane potential information obtained from each node of the ventricular model was converted to phase information in the phase variable state space using Equation 12 (Iyer and Gray, 2001) (mean and standard deviation of PS).

$$\theta(x, y, z, t) = \text{arctan2}\left(\frac{V(x,y,z,t+\tau)-V_{mean}}{V(x,y,z,t)-V_{mean}}\right) \qquad \textbf{Equation 40}$$

where arctan2 is an arctan function considering the quadrant and returns to the phase value from $-\pi$ to $+\pi$. τ is the time delay to calculate the phase of each node and sets as 10 ms, which is the same as the time resolution of the three-dimensional results file from the electrophysiological simulation. V_{mean} refers to the ideal origin. In this study, the mean value of the membrane potentials of all nodes at time t was used as the V_{mean}. We then detected phase singularity by finding the point where the sum of the phase differences in the vicinity became $\pm 2\pi$ (Equation 13). The phase difference at each point was distributed in the range between $(-\pi, \pi)$, as shown in Equation 14. Finally, we counted the number of phase singularity events during ventricular tachyarrhythmia and calculated the mean and the standard deviation of the number of phase singularity to use as the electrical features for predicting the mechanical contractility.

$$sum\ of \Delta\theta = \sum_{n=1}^{4} \Delta\theta_n = \begin{cases} \pm 2\pi, & PS\ point \\ 0, & Otherwise \end{cases} \qquad \textbf{Equation 41}$$

$$\text{Phase Difference (PD)} = \begin{cases} \theta_1 - \theta_2, & |PD| \le \pi \\ \theta_1 - \theta_2 - 2\pi, & |PD| > \pi,\ PD > 0 \\ \theta_1 - \theta_2 + 2\pi, & |PD| > \pi,\ PD < 0 \end{cases} \qquad \textbf{Equation 42}$$

- Filament: Detected by applying the method suggested by Fenton and Karma, which was the method to detect the filament in a three-dimensional cubic mesh, in the 3-dimensional ventricular mesh consisting of tetrahedral elements (Fenton and Karma, 1998, 2002). Equation 15 and Equation 16 are conditions for finding iso-potential points temporally and spatially, respectively. The filament is the line connecting those points satisfied with both of these conditions.

$$\frac{dV_m}{dt} = 0 \qquad \textbf{Equation 43}$$

$$V_m^n = V_m^{n+1} = V_{iso} \qquad \textbf{Equation 44}$$

where V_{iso} refers to the iso-potential and is set from -75 mV to -10 mV, which is the membrane potential range at the phase singularity points detected on the ventricular surface. This considers the property that the filament is internally connected to the phase singularity.

The point that satisfies the spatially iso-potential condition was regarded as the point where the change of potential at the center (p, q, r) for the tetrahedral element is the same.

$$V_m^n = p \cdot V_{x,y,z}^n + q \cdot V_{x+1,y,z}^n + r \cdot V_{x,y+1,z}^n + (1 - p - q - r) \cdot V_{x,y,z+1}^n \quad \textbf{Equation 45}$$

$$V_m^{n+1} = p \cdot V_{x,y,a}^{n+1} + q \cdot V_{x+1,y,z}^{n+1} + r \cdot V_{x,y+1,z}^{n+1} + (1 - p - q - r) \cdot V_{x,y,z+1}^{n+1} \quad \textbf{Equation 46}$$

where p, q, and r can have [0, 1) and set to the 0.5 for a center point in a tetrahedral element. Then, we counted the number of filaments during ventricular tachyarrhythmia and calculated the mean and the standard deviation of filaments.

- The ratio of filament per phase singularity: The filament could be detected therein according to its shape, such as an O-type filament, even though phase singularity was not present (Clayton et al., 2006). To consider the shape and length of filaments, we obtained the ratio of filament per phase singularity and used it as an electrical feature.

Reference

Clayton, R. H., Zhuchkova, E. A., and Panfilov, A. V. (2006). Phase singularities and filaments: Simplifying complexity in computational models of ventricular fibrillation. *Prog. Biophys. Mol. Biol.* 90, 378–398. doi:10.1016/j.pbiomolbio.2005.06.011.

Fenton, F., and Karma, A. (1998). Fiber-rotation-induced vortex turbulence in thick myocardium. *Phys. Rev. Lett.* 81, 481–484. doi:10.1103/PhysRevLett.81.481.

Fenton, F., and Karma, A. (2002). Vortex dynamics in three-dimensional continuous myocardium with fiber rotation: Filament instability and fibrillation. *Chaos An Interdiscip. J. Nonlinear Sci.* 8, 879–879. doi:10.1063/1.166374.

Guccione, J. M., Costa, K. D., and McCulloch, A. D. (1995). Finite element stress analysis of left ventricular mechanics in the beating dog heart. *J. Biomech.* 28, 1167–1177. doi:10.1016/0021-9290(94)00174-3.

Guccione, J. M., and McCulloch, A. D. (1991). Finite Element Modeling of Ventricular Mechanics. *Theory Hear.*, 121–144. doi:10.1007/978-1-4612-3118-9_6.

Gurev, V., Lee, T., Constantino, J., Arevalo, H., and Natalia, A. (2011). Models of Cardiac electromechanics based on individual heart imaging data: image-based electromechanical models of the heart. *Biomech. Model. Mechanobiol.* 10, 295–306. doi:10.1007/s10237-010-0235-5.Models.

Hasegawa, K., Ohno, S., Ashihara, T., Itoh, H., Ding, W. G., Toyoda, F., et al. (2014). A novel KCNQ1 missense mutation identified in a patient with juvenile-onset atrial fibrillation causes constitutively open I Ks channels. *Hear. Rhythm* 11, 67–75. doi:10.1016/j.hrthm.2013.09.073.

Heikhmakhtiar, A. K., Abrha, A. T., Jeong, D. U., and Lim, K. M. (2020). Proarrhythmogenic effect of the L532P and N588K KCNH2 mutations in the human heart using a 3D electrophysiological model. *J. Korean Med. Sci.* 35, 1–13. doi:10.3346/JKMS.2020.35.E238.

Helm, P., Beg, M. F., Miller, M., and Raimond, L. (2005). Measuring and Mapping Cardiac Fiber and Laminar Architecture Using Diffusion Tensor MR Imaging Topics Review of DTMRI and estimation of cardiac fiber. *Ann. N. Y. Acad. Sci.* 1047, 296–307.

Imaniastuti, R., Lee, H. S., Kim, N., Youm, J. B., Shim, E. B., and Lim, K. M. (2014). Computational prediction of proarrhythmogenic effect of the V241F KCNQ1 mutation in human atrium. *Prog. Biophys. Mol. Biol.* 116, 70–75. doi:10.1016/j.pbiomolbio.2014.09.001.

Iyer, A. N., and Gray, R. A. (2001). Experimentalist's approach to accurate localization of phase singularities during reentry. *Ann. Biomed. Eng.* 29, 47–59. doi:10.1114/1.1335538.

Kerckhoffs, R. C. P., Neal, M. L., Gu, Q., Bassingthwaighte, J. B., Omens, J. H., and McCulloch, A. D. (2007). Coupling of a 3D finite element model of cardiac ventricular mechanics to lumped systems models of the systemic and pulmonic circulation. *Ann. Biomed. Eng.* 35, 1–18. doi:10.1007/s10439-006-9212-7.

Kharche, S., Adeniran, I., Stott, J., Law, P., Boyett, M. R., Hancox, J. C., et al. (2012). Pro-arrhythmogenic effects of the S140G KCNQ1 mutation in human atrial fibrillation - insights from modeling. *J. Physiol.* 590, 4501–4514. doi:10.1113/jphysiol.2012.229146.

Ki, C. S., Jung, C. L., Kim, H. J., Baek, K. H., Park, S. J., On, Y. K., et al. (2014). A KCNQ1 mutation causes age-dependent bradycardia and persistent atrial fibrillation. *Pflugers Arch. Eur. J. Physiol.* 466, 529–540. doi:10.1007/s00424-013-1337-6.

Loewe, A., Wilhelms, M., Fischer, F., Scholz, E. P., Dössel, O., and Seemann, G. (2014). Arrhythmic potency of human ether-à-go-go-related gene mutations L532P and N588K in a computational model of human atrial myocytes. *Europace* 16, 435–443. doi:10.1093/europace/eut375.

Ng, J., Kadish, A. H., and Goldberger, J. J. (2006). Effect of electrogram characteristics on the relationship of dominant frequency to atrial activation rate in atrial fibrillation. *Hear. Rhythm* 3, 1295–1305. doi:10.1016/j.hrthm.2006.07.027.

Rice, J. J., Wang, F., Bers, D. M., and De Tombe, P. P. (2008). Approximate model of cooperative activation and crossbridge cycling in cardiac muscle using ordinary differential equations. *Biophys. J.* 95, 2368–2390. doi:10.1529/biophysj.107.119487.

Ten Tusscher, K. H. W. J. (2004). A model for human ventricular tissue. *AJP Hear. Circ. Physiol.* 286, H1573–H1589. doi:10.1152/ajpheart.00794.2003.

Ten Tusscher, K. H. W. J., and Panfilov, A. V. (2006). Alternans and spiral breakup in a human ventricular tissue model. *Am. J. Physiol. Circ. Physiol.* 291, H1088–H1100. doi:10.1152/ajpheart.00109.2006.

Usyk, T. P., LeGrice, I. J., and McCulloch, A. D. (2002). Computational model of three-dimensional cardiac electromechanics. *Comput. Vis. Sci.* 4, 249–257. doi:10.1007/s00791-002-0081-9.

Yuniarti, A. R., Setianto, F., Marcellinus, A., Hwang, H. J., Choi, S. W., Trayanova, N., et al. (2018). Effect of KCNQ1 G229D mutation on cardiac pumping efficacy and reentrant dynamics in ventricles: Computational study. *Int. j. numer. method. biomed. eng.* 34, 1–16. doi:10.1002/cnm.2970.

Supplementary Figures

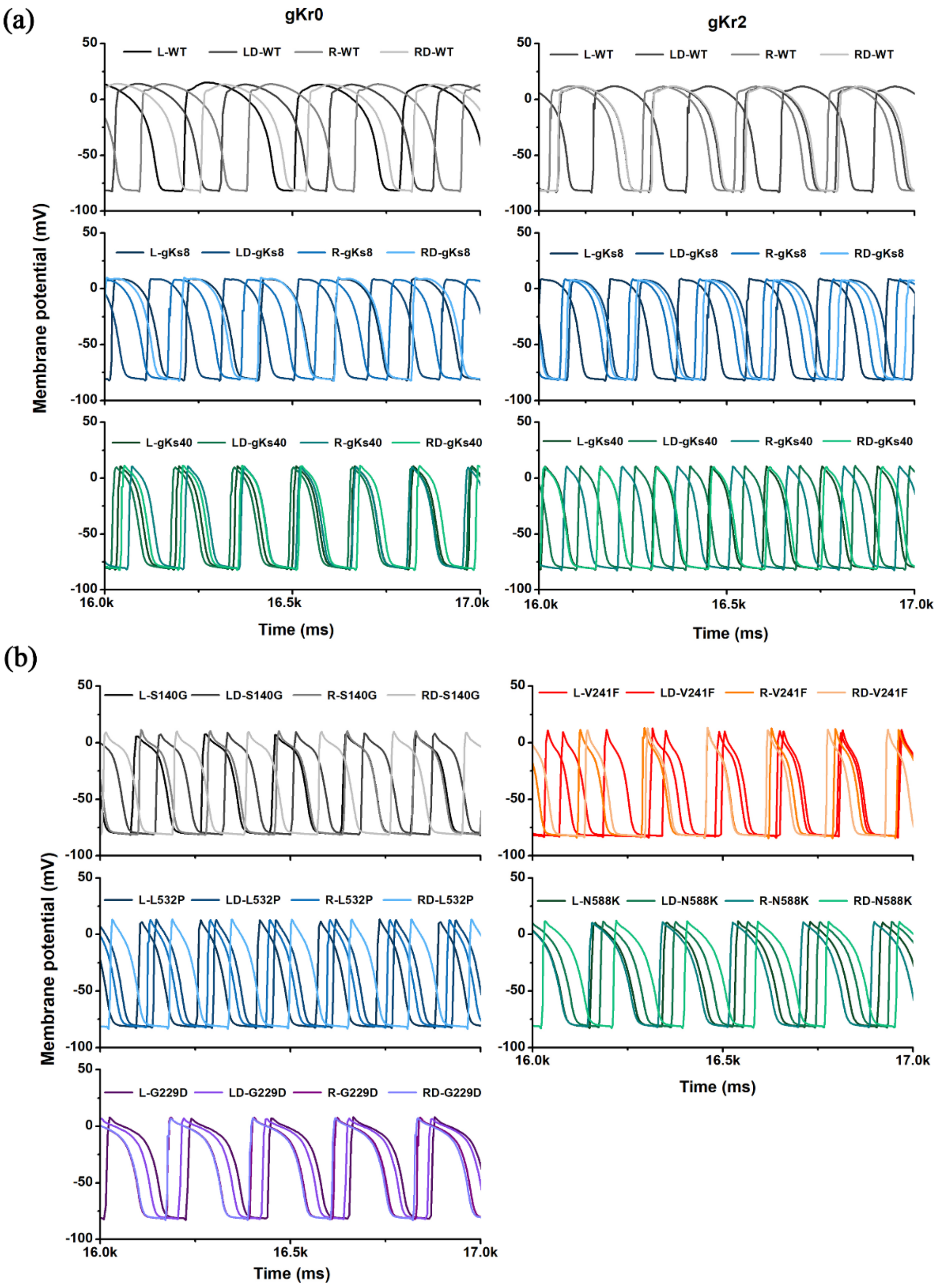

Supplementary Figure S 1. Action potential shapes from electrophysiological simulations. (a), action potential shapes according to the g_{Ks} variations; (b), action potential shape according to the mutation conditions.

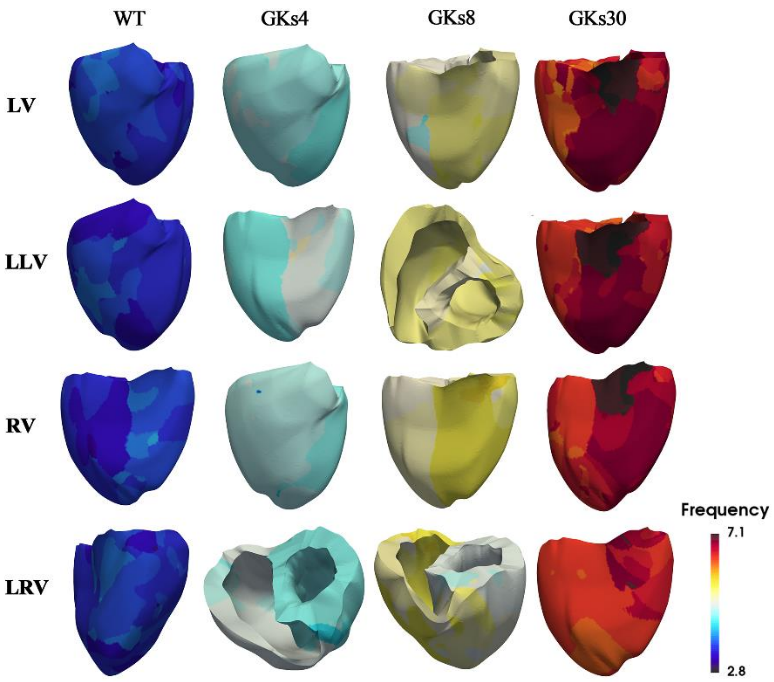

(a)

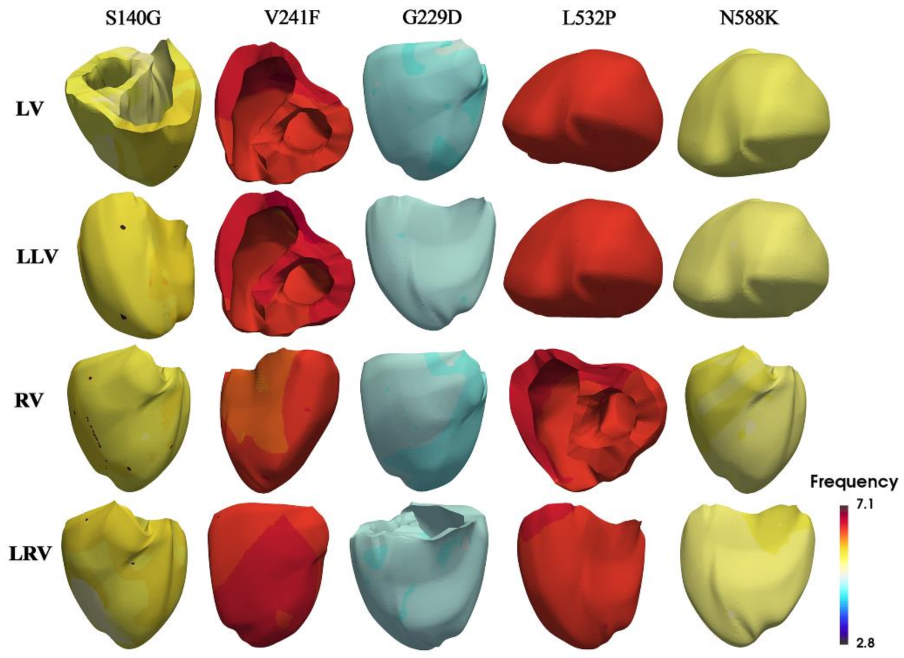

(b)

Supplementary Figure S 2. Contours for dominant frequency. (a), dominant frequency contours according to the g_{Ks} variations; (b), dominant frequency contours according to the mutation conditions.

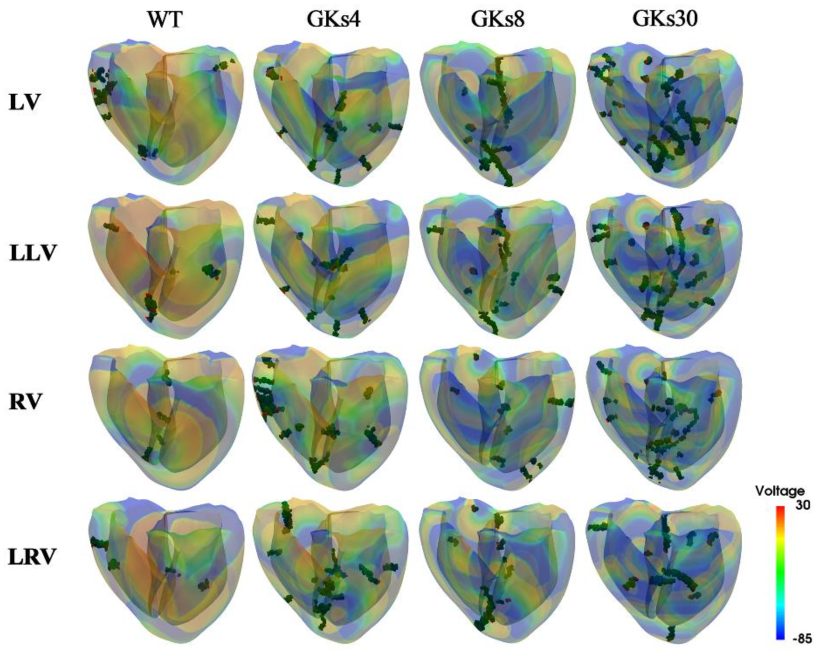

(a)

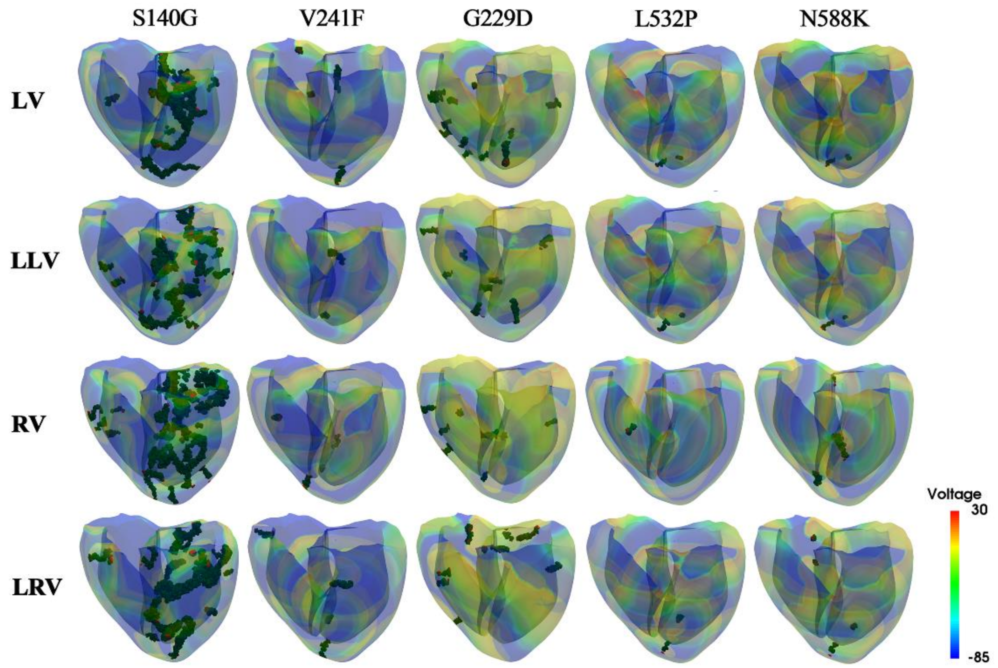

(b)

Supplementary Figure S 3. Membrane voltage contour map with phase singularities and filaments. (a), contours according to the g_{Ks} variations; (b), contours according to the mutation conditions.

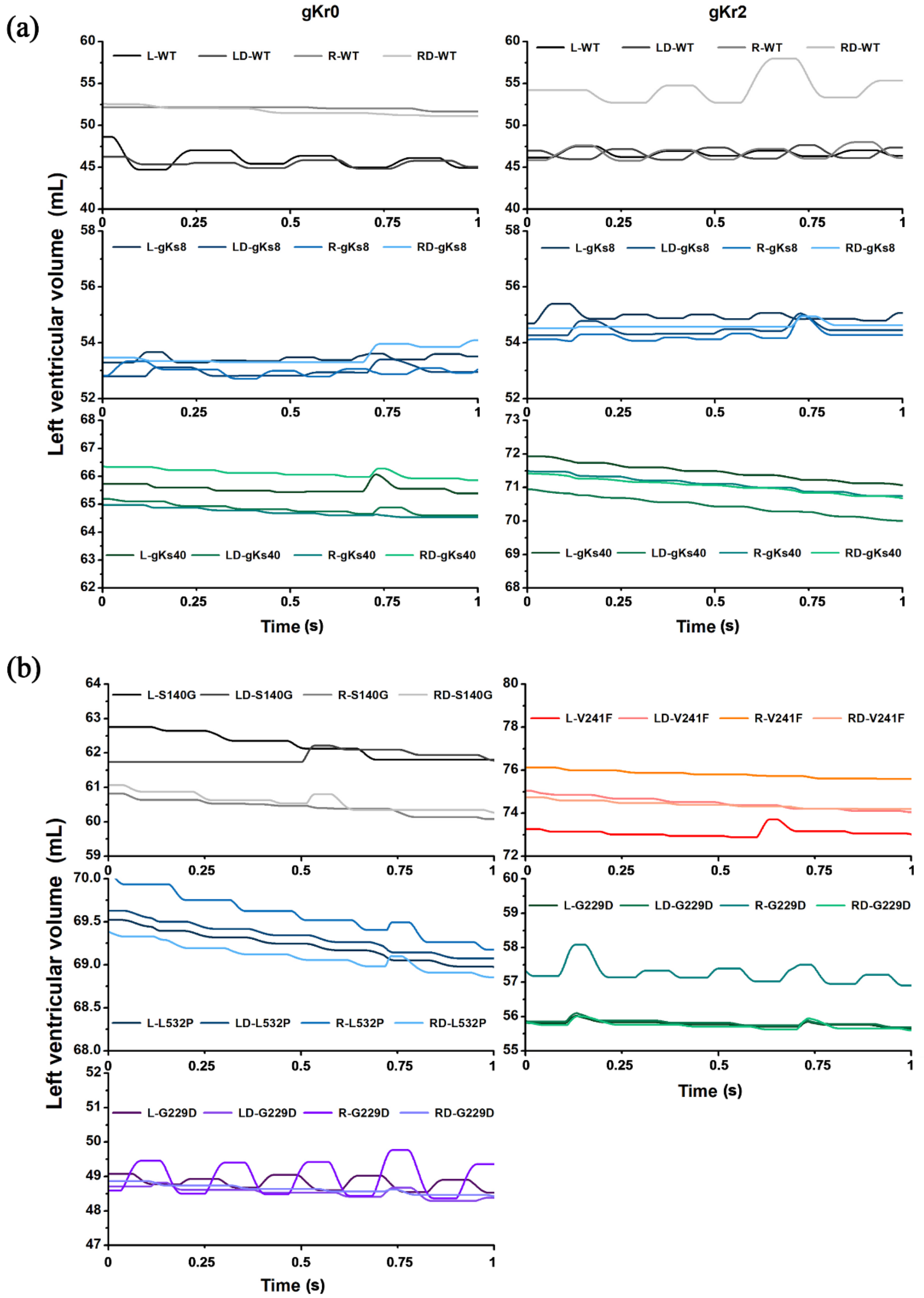

Supplementary Figure S 4. Left ventricular volume curves during ventricular fibrillation. (a), according to the g_{Ks} variations; (b), according to the mutation conditions.

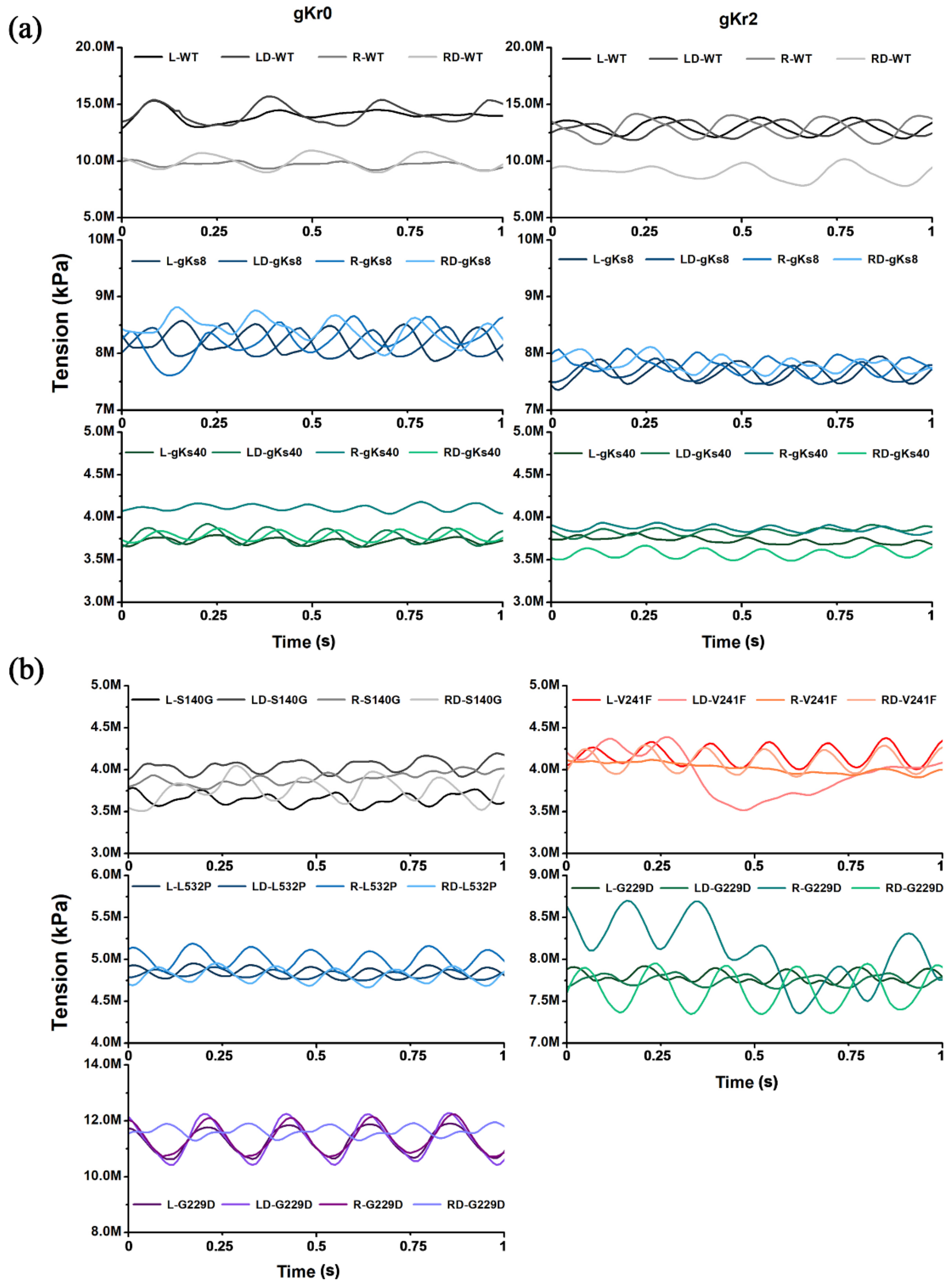

Supplementary Figure S 5. Myocardial tension curves during ventricular fibrillation. (a), according to the g_{Ks} variations; (b), according to the mutation conditions.

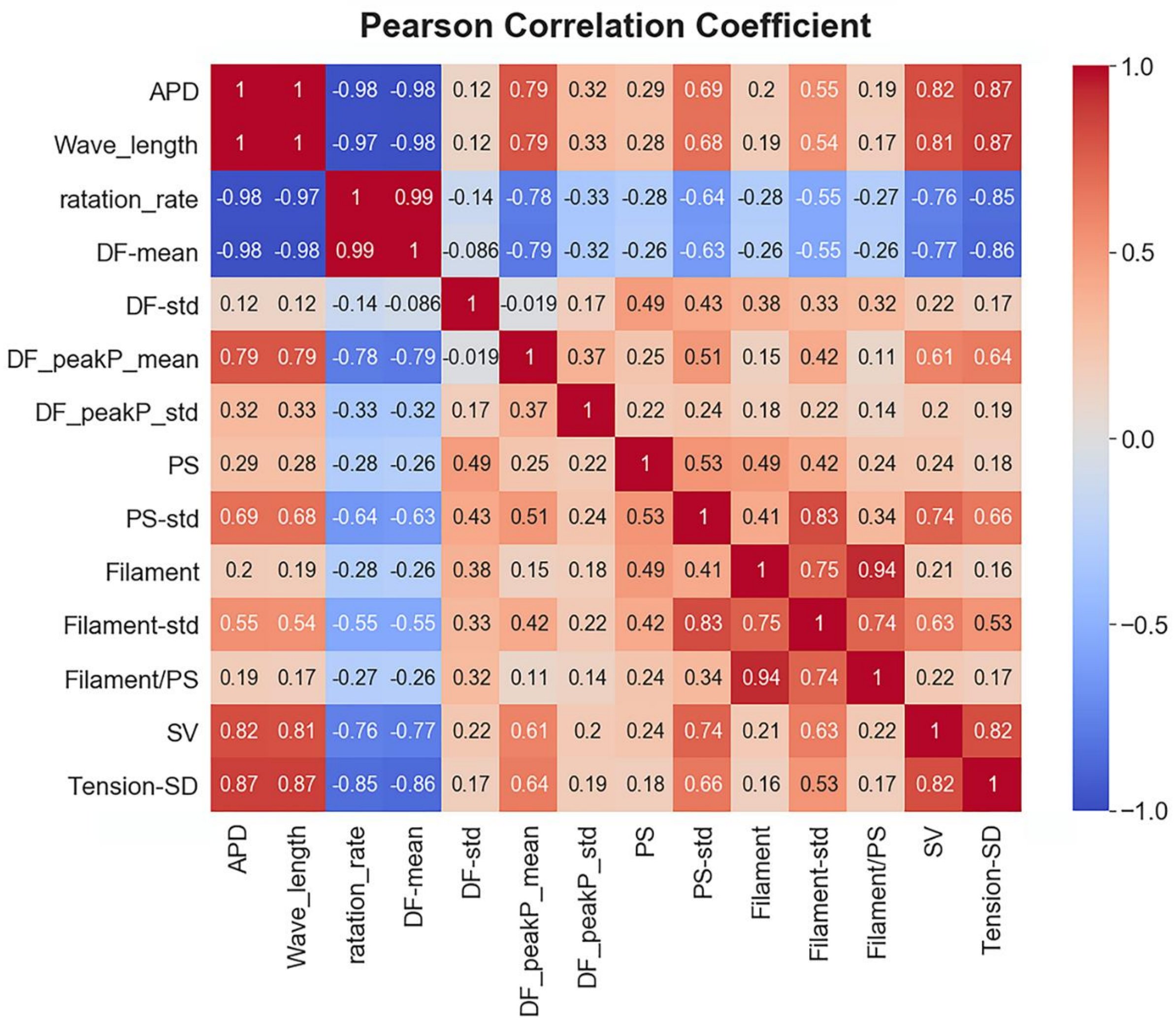

Supplementary Figure S 6. Pearson correlation coefficient of electrical parameters and mechanical parameters. APD, action potential duration; DF, dominant frequency; PS, the number of phase singularities; Filament, the number of filaments; SV, stroke volume; ampTens, the amplitude of myocardial tension.

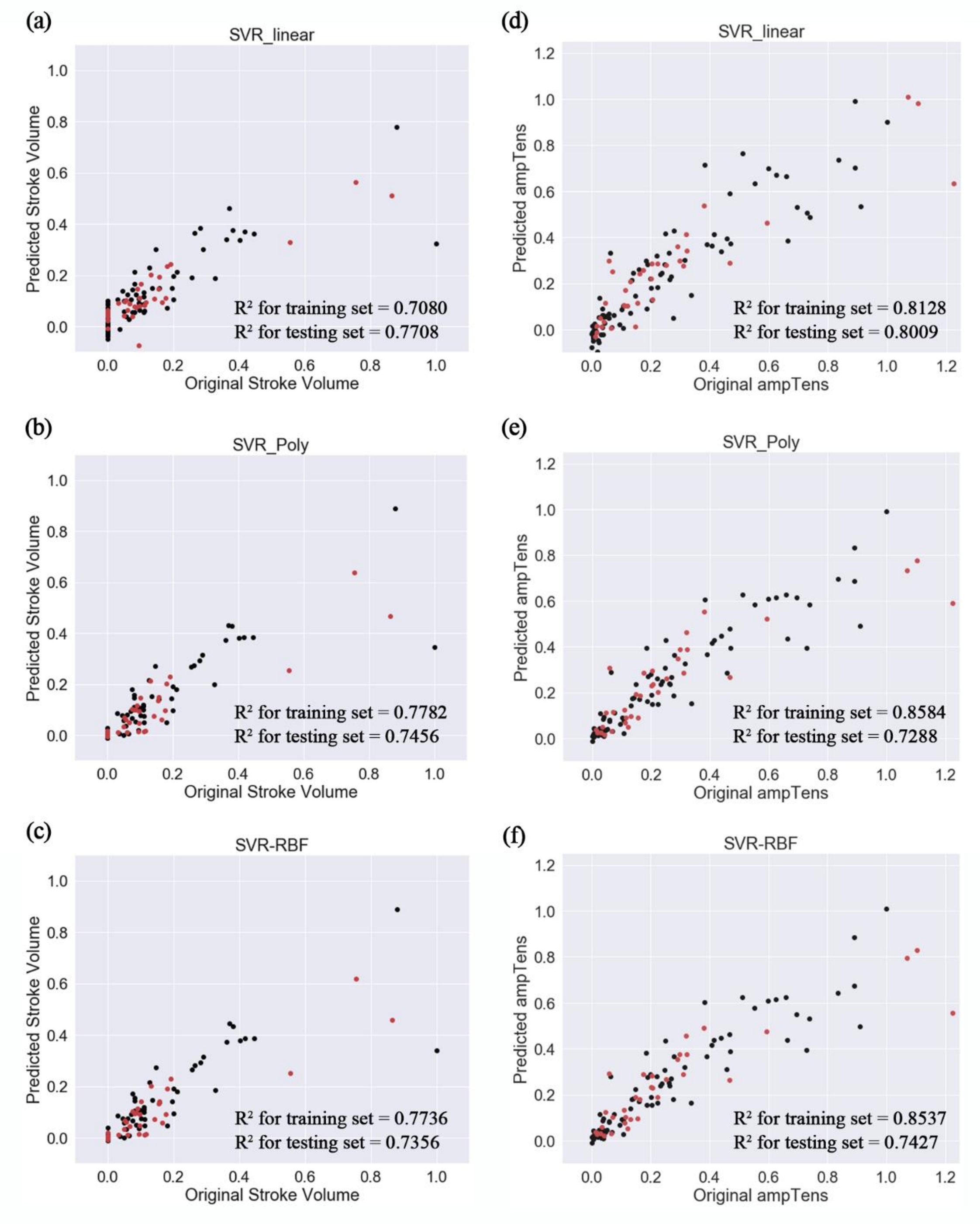

Supplementary Figure S 7. Accuracies of support vector regression using filtered features. Accuracies of stroke volume prediction using SVR with linear (a), polynomial (b), and RBF kernels (c); Accuracies of ampTens prediction using SVR with linear (d), polynomial (e), and RBF kernels (f), respectively

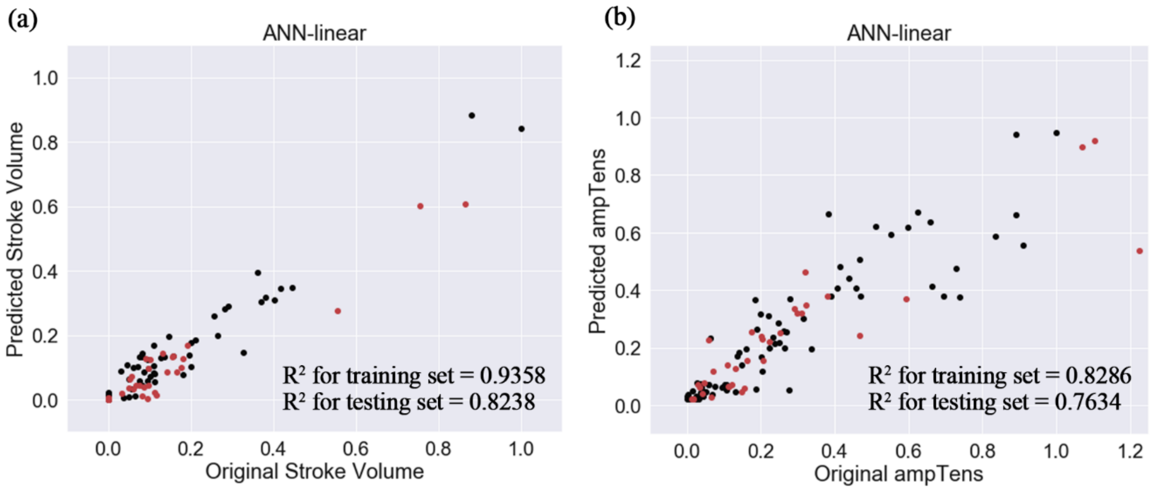

Supplementary Figure S 8. Accuracies of artificial neural network regression models with 4-hidden layers. (**a**) Prediction of stroke volume, (**b**) Prediction of myocardial tension (ampTens)
